# Supplementary material for: NS2B-D55E and NS2B-E65D Variations Are Responsible for Differences in NS2B-NS3 Protease Activities Between Japanese Encephalitis Virus Genotype I and III in Fluorogenic Peptide Model
Source: Int J Mol Sci. 2024 Nov 26;25(23):12680. doi: 10.3390/ijms252312680 (PMC11641239; doi:10.3390/ijms252312680)
Supplement: Supplementary file 1 [file ijms-25-12680-s001.zip › ijms-3290794-supplementary.pdf]

Supplementary Figure# S1

A)

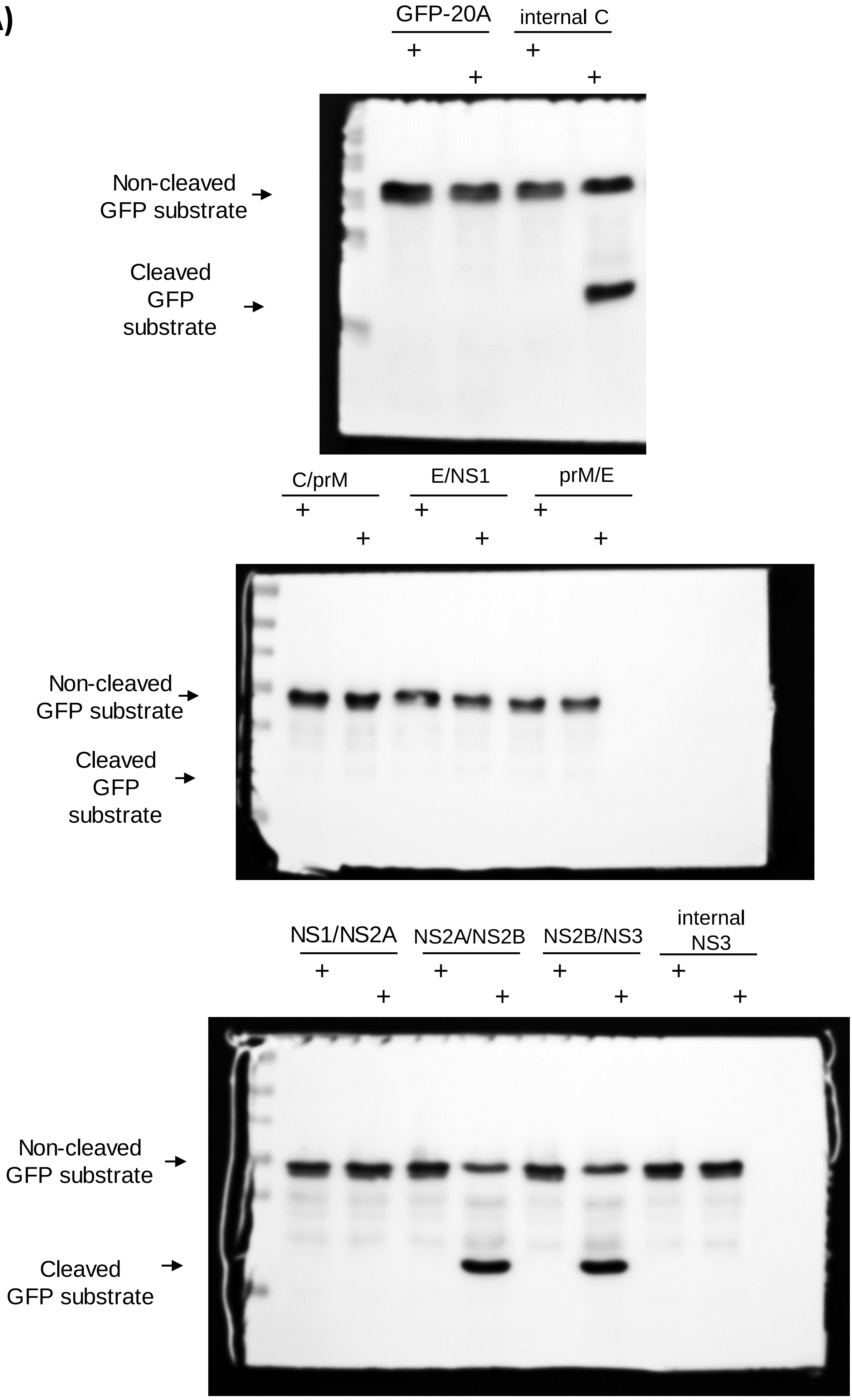

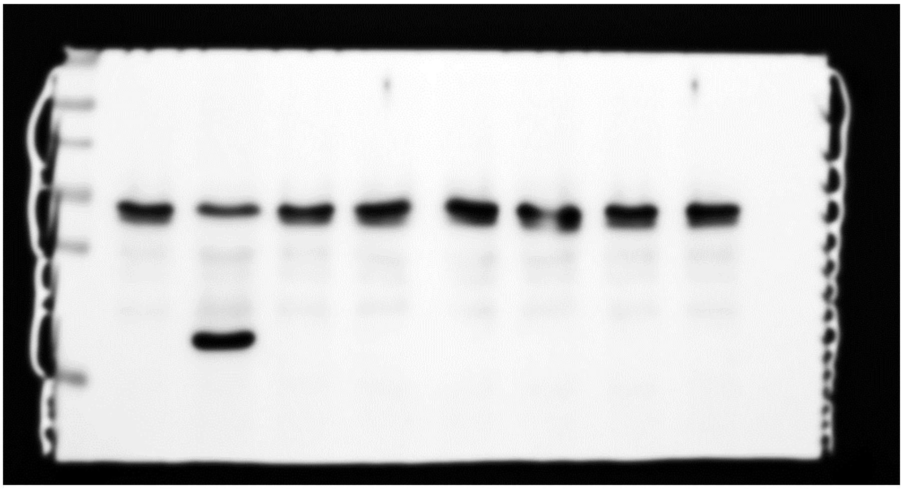
NS3/NS4A
internal NS4A
NS4A/NS4B
NS4B/NS5
+
+
+
+
+
+
+
+
Non-cleaved GFP substrate
Cleaved GFP substrate

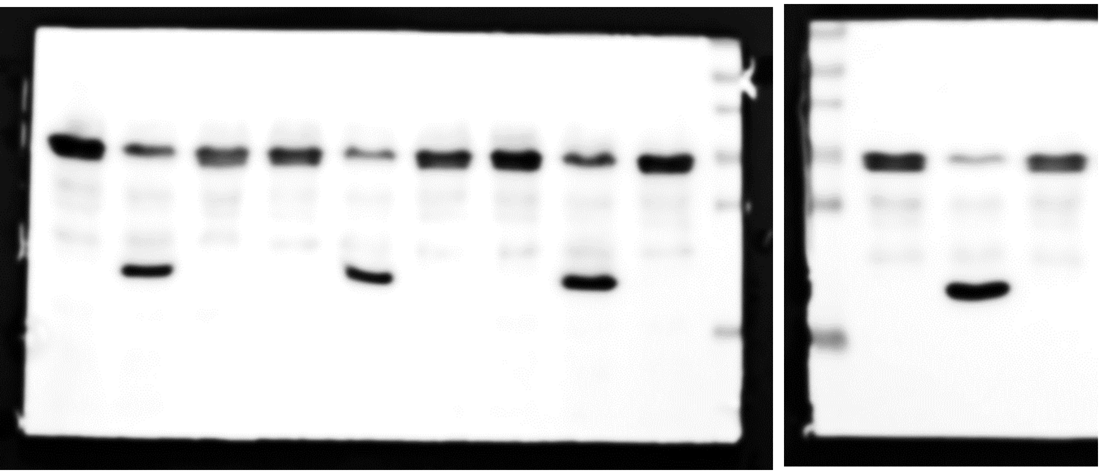
GFP-substrate
GFP-substrate+NS2B(H)-NS3(pro)
GFP-substrate+NS2B(H)-NS3(pro)-dead
GFP-substrate
GFP-substrate+NS2B(H)-NS3(pro)
GFP-substrate+NS2B(H)-NS3(pro)-dead
GFP-substrate
GFP-substrate+NS2B(H)-NS3(pro)
GFP-substrate+NS2B(H)-NS3(pro)-dead
GFP-substrate
GFP-substrate+NS2B(H)-NS3(pro)
GFP-substrate+NS2B(H)-NS3(pro)-dead
Non-cleaved GFP substrate
Cleaved GFP substrate
internal C
NS2A/NS2B
NS2B/NS3
NS3/NS4A

B)

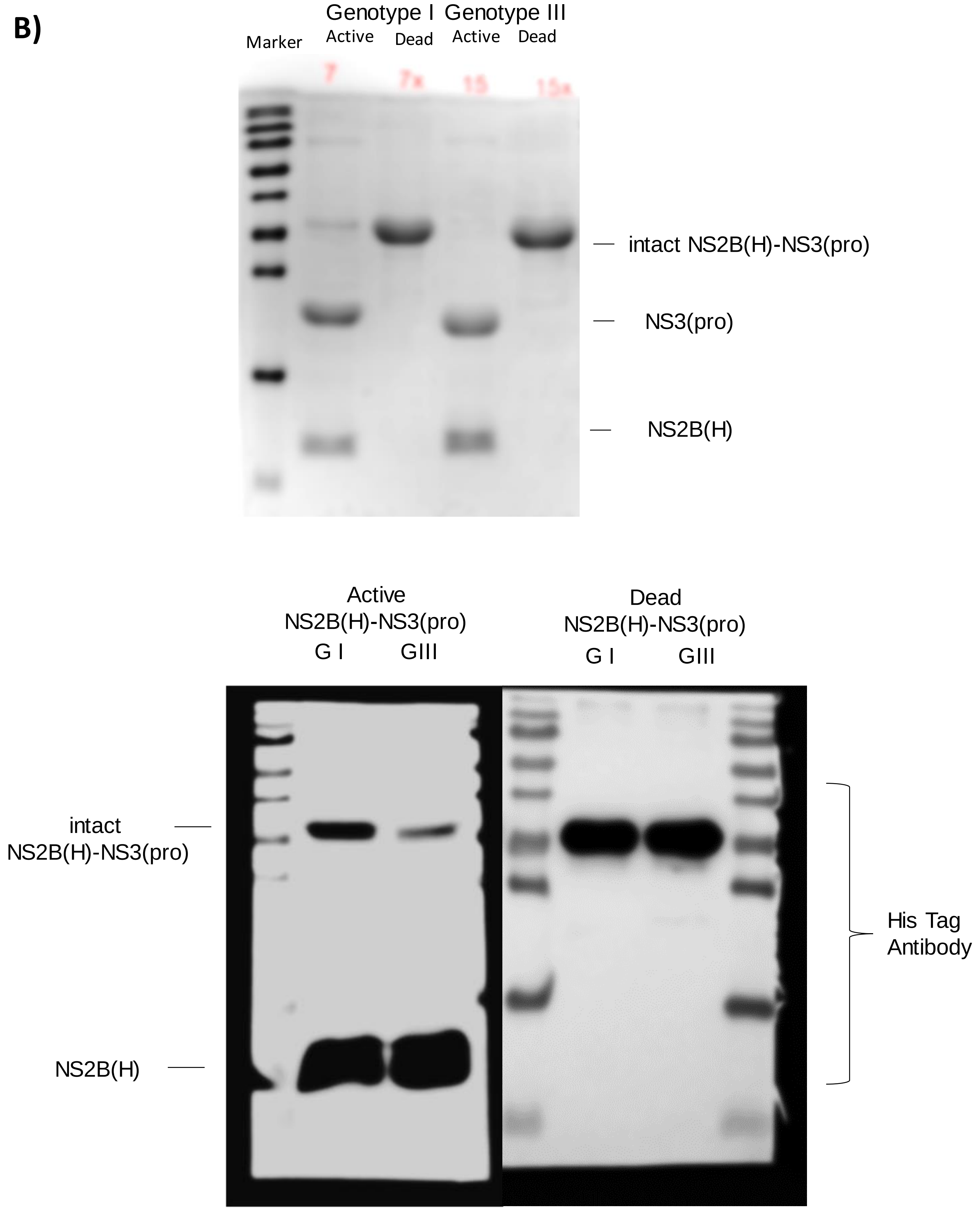

Supplementary Figure# S1. Original Blots. **A)** Blots from Figure 2 in main manuscript . **B)** Blots from Figure 3 in main manuscript.
